# Supplementary material for: Genetic accessibility, not superior fitness, determines which anti-HBs antibody escape variants are selected in patients
Source: bioRxiv. 2026 Oct 7:2026.10.06.756817. Preprint. [Version 1] doi: 10.64898/2026.10.06.756817 (PMC13692201; doi:10.64898/2026.10.06.756817)
Supplement: Supplementary file 1 [file NIHPP2026.10.06.756817v1-supplement-1.pdf]

bioRxiv preprint doi: https://doi.org/10.64898/2026.10.06.756817; this version posted October 8, 2026. The copyright holder for this preprint (which was not certified by peer review) is the author/funder, who has granted bioRxiv a license to display the preprint in perpetuity. It is made available under a CC-BY 4.0 International license.

Sup Figure S1: launching HBV isolates in huFNRG mice

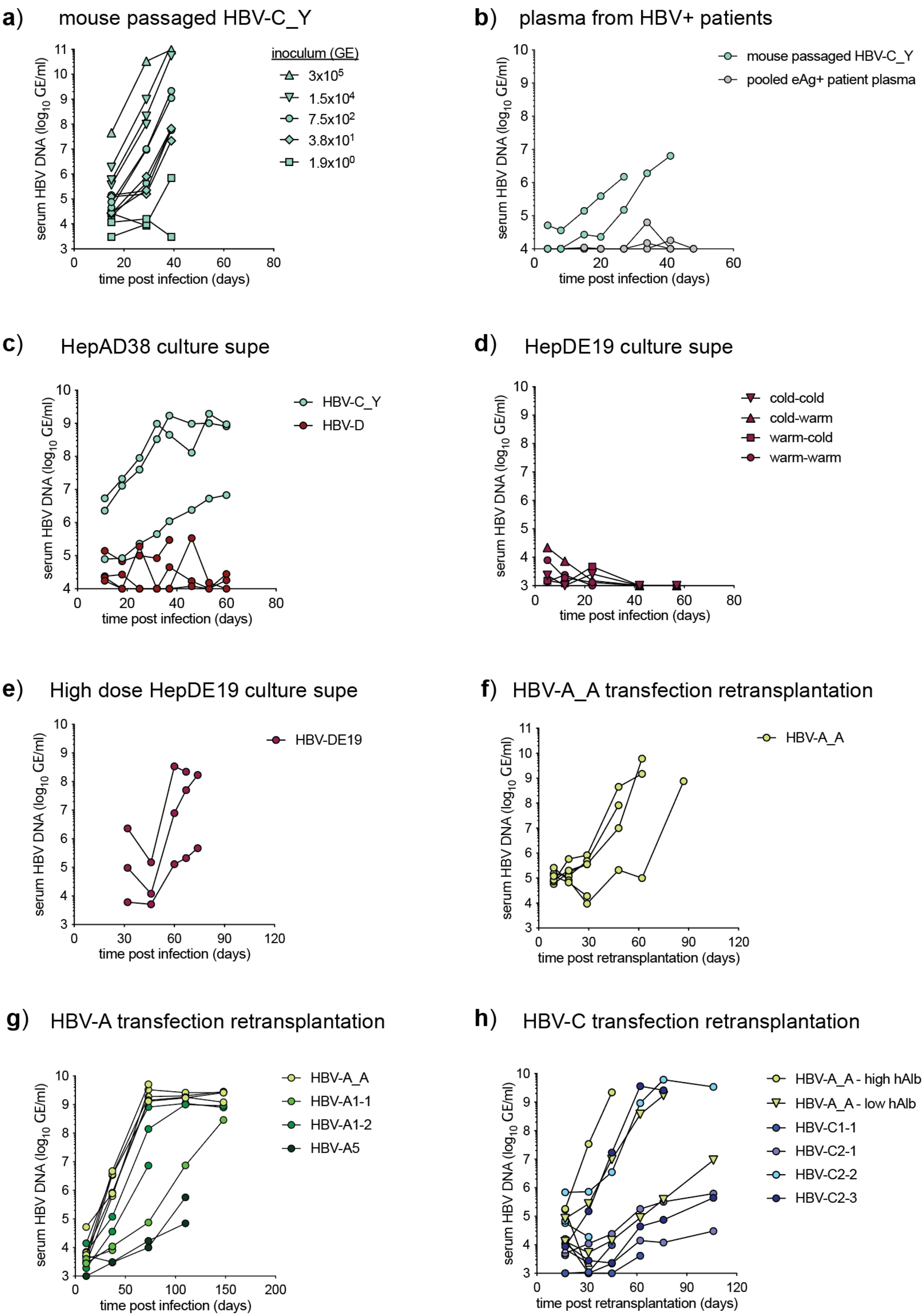

bioRxiv preprint doi: https://doi.org/10.64898/2026.10.06.756817; this version posted October 8, 2026. The copyright holder for this preprint (which was not certified by peer review) is the author/funder, who has granted bioRxiv a license to display the preprint in perpetuity. It is made available under a CC-BY 4.0 International license.

Sup Figure S2: Silent mutants to quantify DNA launch efficiencies

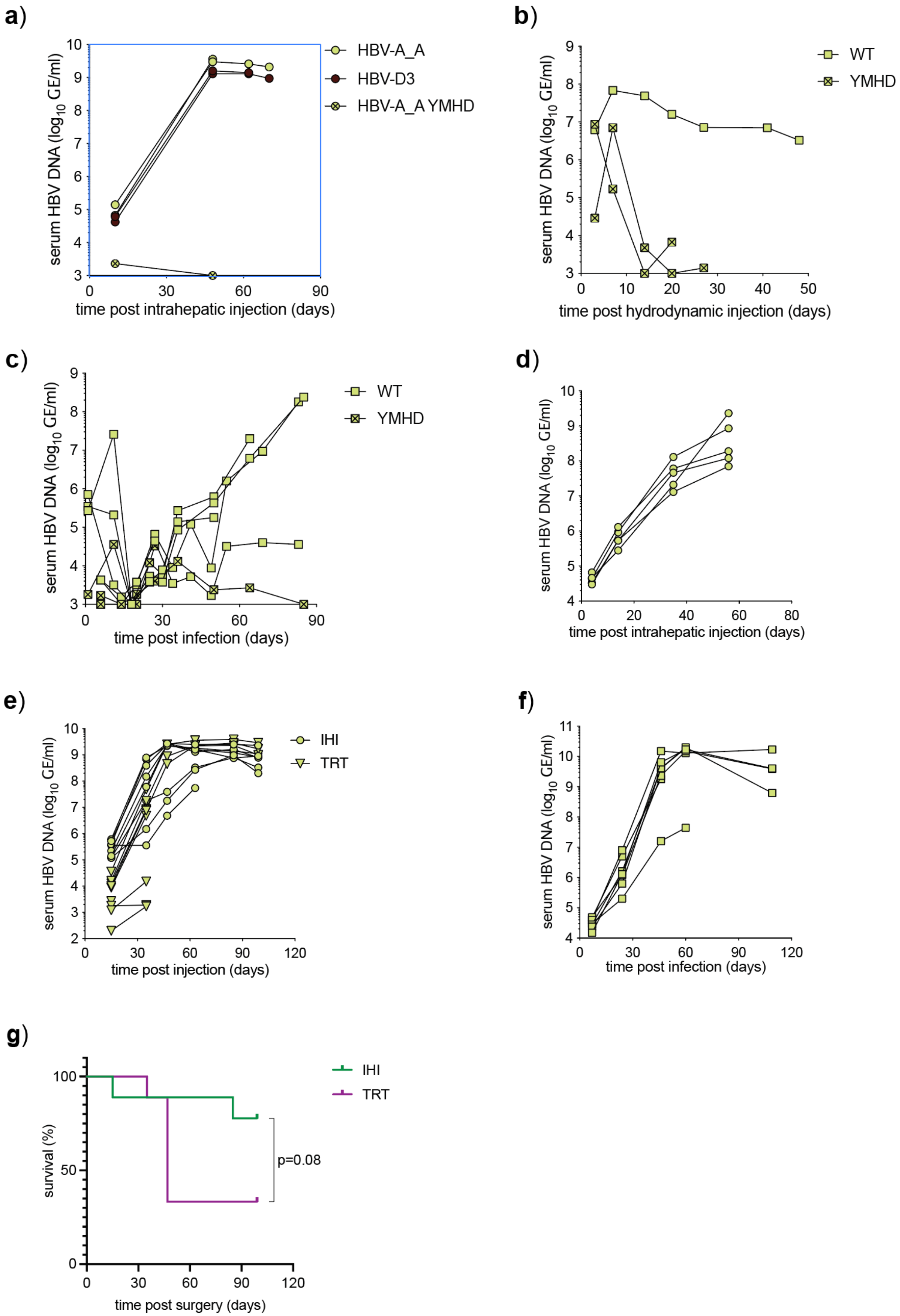

bioRxiv preprint doi: https://doi.org/10.64898/2026.10.06.756817; this version posted October 8, 2026. The copyright holder for this preprint (which was not certified by peer review) is the author/funder, who has granted bioRxiv a license to display the preprint in perpetuity. It is made available under a CC-BY 4.0 International license.

Sup Figure S3: Comparing DNA launch efficiecies across methods

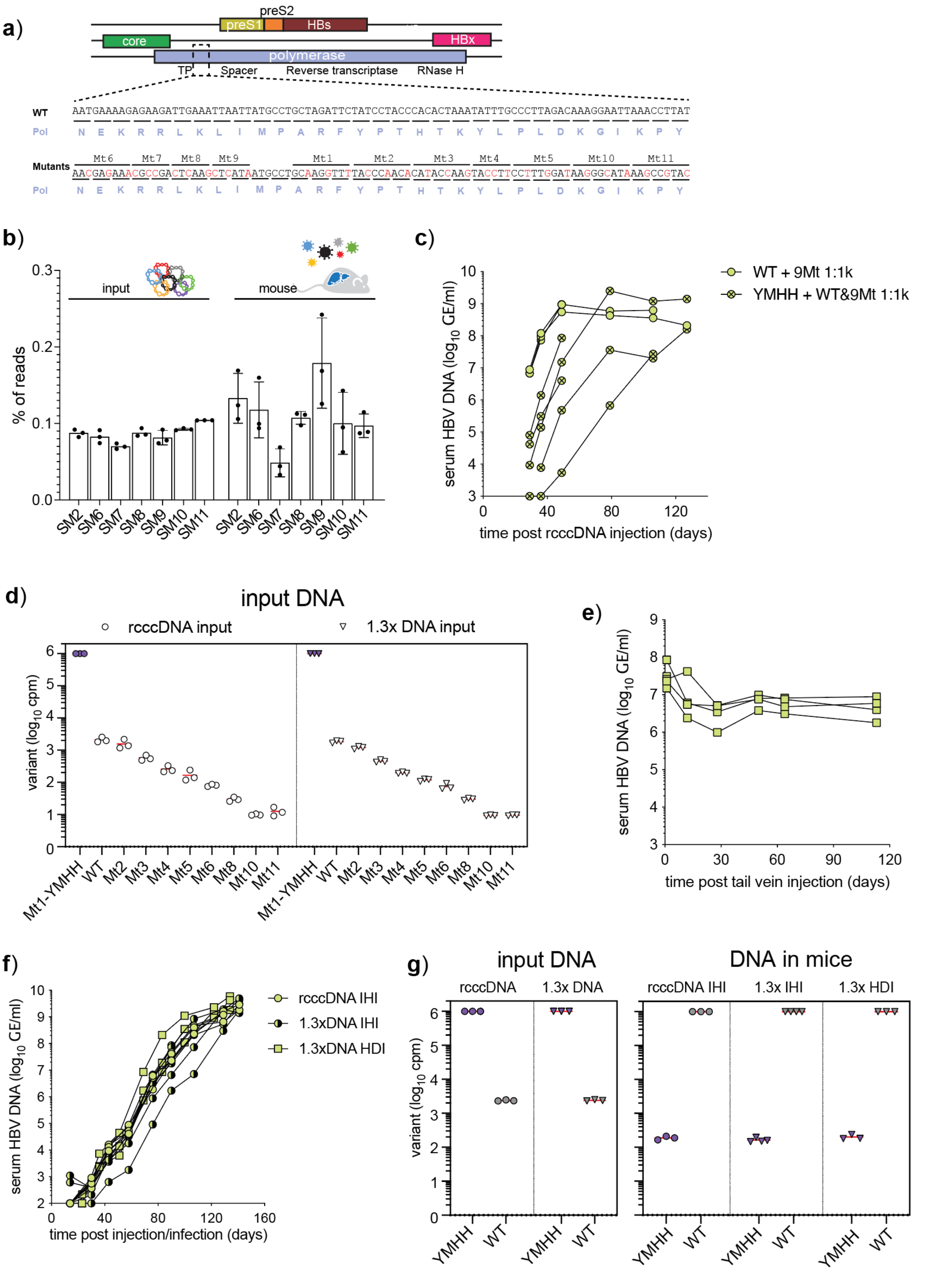

bioRxiv preprint doi: https://doi.org/10.64898/2026.10.06.756817; this version posted October 8, 2026. The copyright holder for this preprint (which was not certified by peer review) is the author/funder, who has granted bioRxiv a license to display the preprint in perpetuity. It is made available under a CC-BY 4.0 International license.

Sup Figure S4: Identification of HBs neutralizing antibody resistant variants using deep mutational scanning

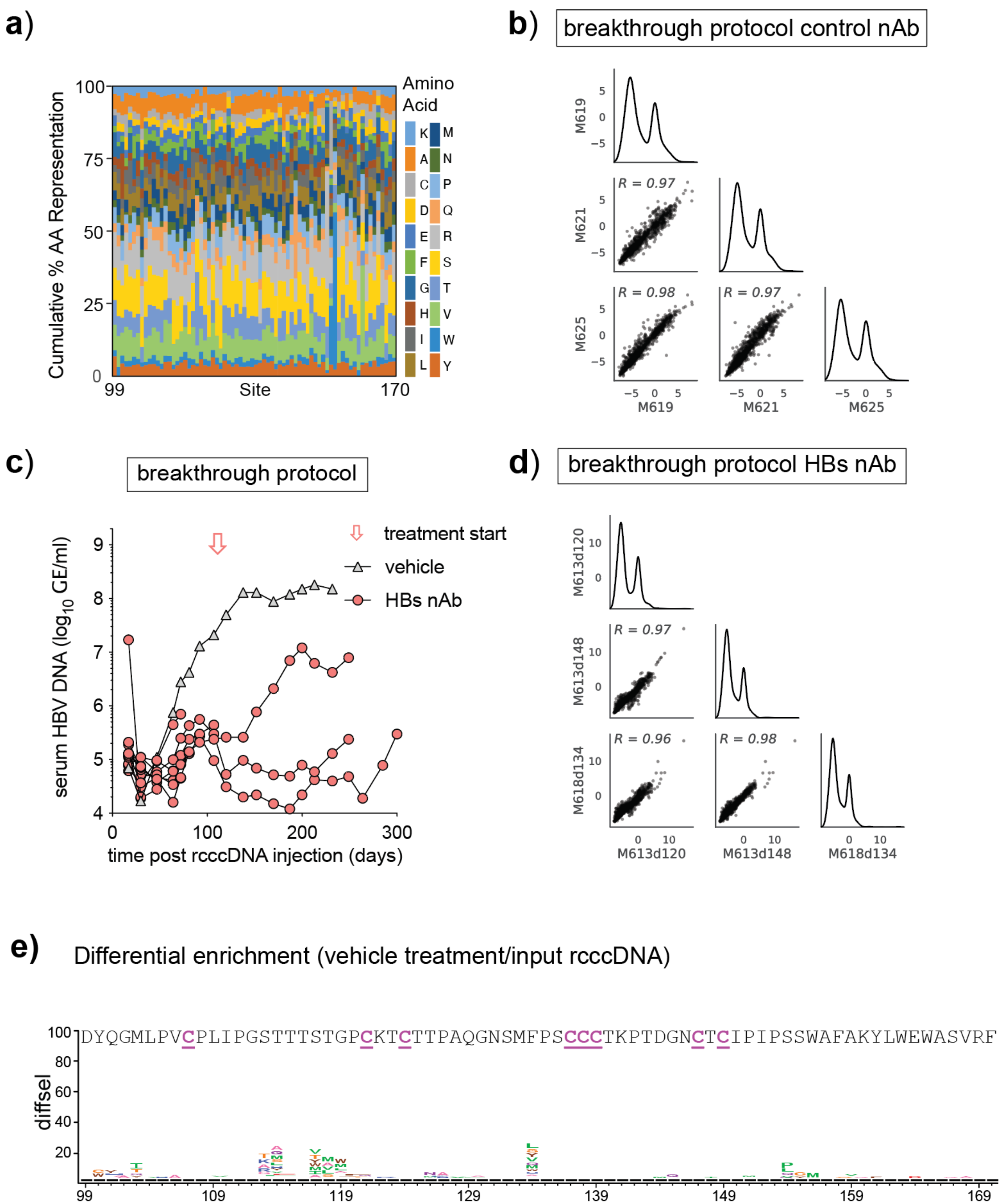

bioRxiv preprint doi: https://doi.org/10.64898/2026.10.06.756817; this version posted October 8, 2026. The copyright holder for this preprint (which was not certified by peer review) is the author/funder, who has granted bioRxiv a license to display the preprint in perpetuity. It is made available under a CC-BY 4.0 International license.

Sup Figure legends

Sup Figure S1: Launching HBV isolates in huFNRG mice

a) huFNRG mice were challenged intravenously with HBV-C_Y containing serum at 20-fold dilutions spanning an inoculum range from $1.9x10^0$ to $3x10^5$ genome equivalent (GE) per mouse, and HBV DNA in mouse serum was quantified over time. All mice in the $3.8x10^1$ GE group and 1 of 3 mice in the $1.9x10^0$ GE group became viremic.

b) huFNRG mice were challenged intravenously with pooled plasma from four $HBeAg^+$ blood donors at $>5×10^8$ GE per donor, or mouse-passaged HBV-C_Y serum. None of these four patient isolates established viremia over this time course.

c) huFNRG mice were challenged intravenously with cell culture supernatant from HepAD38 cells containing $4×10^6$ or $2×10^8$ GE, or mouse-passaged HBV-C_Y serum. HepAD38 supernatants did not induce viremia over this time course, regardless of dose.

d) Culture supernatants from HepDE19 cells were stored at 37°C or at 4°C prior to injection of $1×10^8$ GE into huFNRG mice. This did not result in viremia over this time course.

e) Culture supernatants from HepDE19 cells were concentrated using polyethylene glycol prior to injection of $3×10^9$ GE per huFNRG mouse. After an initial decline, all mice became viremic.

f) mpPHH cultures were transfected with HBV-A_A rcccDNA prior to retransplantation into huFNRG mice. Four of five mice became viremic.

g-h) The transfection and retransplantation protocol was used to launch different consensus sequences from HBV-A and HBV-C subgenotypes, showing variable success between and within isolates (high hAlb = 10 mg/ml, low hAlb = 2 mg/ml).

For **a-h**) symbols represent individual mice.

Sup Figure S2: Silent mutants to quantify launch methods

a) huFNRG mice underwent IHI with HBV-A_A, HBV-D3 or a polymerase dead (YMHD) HBV-A_A isolate. HBV DNA in mouse serum was quantified over time (symbols represent individual mice).

b) NRG mice underwent hydrodynamic tail vein injection with WT or catalytically dead polymerase (YMHD) 1.3x HBV plasmid DNA and HBV DNA was quantified over time (symbols represent individual mice).

c) huFNRG mice were challenged with serum from NRG mice 21 days after hydrodynamic tail vein injection containing $7x10^5$ GE/mouse (symbols represent individual mice).

d) An equal ratio of HBV-A_A WT, Mt1-5, and polymerase dead (YMHH) control rcccDNA was launched in huFNRG mice through IHI. HBV DNA in mouse serum was quantified over time (symbols represent individual mice).

e) HBV DNA in mouse serum was quantified over time following launch using the following mixture: Mt2-5 were diluted 10-fold into WT rcccDNA, starting with Mt2 at 1:10 and ending with Mt5 at 1:10,000. This was

bioRxiv preprint doi: https://doi.org/10.64898/2026.10.06.756817; this version posted October 8, 2026. The copyright holder for this preprint (which was not certified by peer review) is the author/funder, who has granted bioRxiv a license to display the preprint in perpetuity. It is made available under a CC-BY 4.0 International license.

transfected into mpPHH prior to retransplantation (TRT) or delivered through IHI (symbols represent individual mice).

f) huFNRG mice were infected with serum from NRG mice that underwent HDI with 1.3x plasmid DNA containing Mt2-5 at a 10-fold dilution similar to **e**). HBV DNA in mouse serum was quantified over time (symbols represent individual mice).

g) Groups of nine huFNRG mice underwent intrahepatic rcccDNA injection (IHI) or mpPHH transfection and retransplantation (TRT) and were monitored for survival (Mantel-Cox test).

Sup Figure S3: Comparing DNA launch efficiencies across methods

a) HBV genome organization with overlapping open reading frames shown in color with nucleotide changes for Mutants (Mt) 1–11 highlighted in red.

b) An additional 6 mutants (Mt6-11) in the TP domain were spiked into HBV-A_A WT rcccDNA at 1:1,000 and launched using rcccDNA IHI, using silent Mt2 as a positive control. At peak viremia, all mutants, except Mt7 and Mt9, were present at approximately the same frequency as Mt2 and deemed phenotypically silent (input symbols represent technical triplicates, mouse symbols represent individual mice).

c) huFNRG mice underwent IHI with HBV-A_A WT rcccDNA or YMHH carrier rcccDNA with WT rcccDNA spiked in at 1:1,000. YMHH carrier rcccDNA resulted in delayed viremia kinetics compared to WT rcccDNA (symbols represent individual mice).

d) Amplicon sequencing of rcccDNA and 1.3× input DNA with the silent mutants spiked into YMHH rcccDNA carrier DNA starting at 1:1,000 at 2-fold dilutions showed the expected dilutions in both input samples (cpm = counts per million, symbols represent technical triplicates).

e) NRG mice underwent HDI with WT and 8 silent mutants diluted into catalytically dead polymerase (YMHH)1.3x HBV plasmid "carrier" DNA starting at 1:1000 and then 2-fold down (symbols represent individual mice).

f) huFNRG mice were infected with silent mutant NRG serum or underwent IHI with WT and 8 silent mutant rcccDNA spiked into YMHH carrier DNA, starting with WT at 1:1000 and silent mutants at 2-fold serial dilution. HBV DNA in mouse serum was quantified over time (symbols represent individual mice).

g) YMDD (WT) region amplicon sequencing of input DNA and mice at peak viremia (cpm = counts per million, input symbols represent technical triplicates, mouse symbols are individual mice).

Sup Figure S4: Identification of HBs neutralizing antibody-resistant variants

a) Amino acid representation across the codon-randomized library.

Stacked bar plot showing the cumulative percentage of non-wildtype amino acid representation at each site. Codon counts were averaged across triplicate input DNA libraries, aggregated by amino acid, and normalized after removing the wild-type amino acid at each position. The resulting plot illustrates the relative composition of variant amino acids across the scanned region.

bioRxiv preprint doi: https://doi.org/10.64898/2026.10.06.756817; this version posted October 8, 2026. The copyright holder for this preprint (which was not certified by peer review) is the author/funder, who has granted bioRxiv a license to display the preprint in perpetuity. It is made available under a CC-BY 4.0 International license.

b) Reproducibility of control nAb LY1404 differential selection measurements across mice. Selection values were calculated using dms_tools2 and compared across amplicons covering the DMS region, amplified from serum-derived DNA obtained from three individual mice (M619, M621, M625) on day 148 post inoculation with 1xrcccDNA. Each mouse was analyzed with three technical replicates derived from the same amplicon obtained from the input 1xrcccDNA library. Each point represents a single amino-acid mutation. Pearson correlation coefficients are shown for each pairwise comparison.

c) Repeat breakthrough protocol in huFNRG mice with serum hAlb levels between 4.4 and 15 mg/ml (median 7.7 mg/ml). HBs nAb treatment was initiated on day 111 after IHI, while one mouse near peak viremia received vehicle. Virus in one of the three mice escaped HBs nAb treatment, while the remaining two mice died before reaching viremia levels where variants could be quantified.

d) Reproducibility of HBs nAb HBc differential selection measurements across samples. Selection values were calculated using dms_tools2 and compared across amplicons covering the DMS region, amplified from serum-derived DNA obtained from three samples from two mice (M613, day 120 and day 148 post inoculation; and M618, day 148 post inoculation with 1xrcccDNA). Each sample was analyzed with three technical replicates derived from the same amplicon obtained from the input 1xrcccDNA library. Each point represents a single amino-acid mutation. Pearson correlation coefficients are shown for each pairwise comparison.

e) Amino acid logoplot of differential enrichment in the AGL without antibody treatment. The WT amino acid sequence is shown on top, highlighting the eight cysteine residues that form disulfide bonds. The overall pattern of enrichment and regions lacking substitutions is similar to that observed in mice treated with the control nAb, with differences around amino acid 109 (diffsel = $\log_2$ enrichment relative to the input 1xrcccDNA library).

bioRxiv preprint doi: https://doi.org/10.64898/2026.10.06.756817; this version posted October 8, 2026. The copyright holder for this preprint (which was not certified by peer review) is the author/funder, who has granted bioRxiv a license to display the preprint in perpetuity. It is made available under a CC-BY 4.0 International license.

Supplemental Table 1: Abbreviations

AGL	antigenic loop
DMS	deep mutational scanning
GE	genome equivalents
HBV	hepatitis B virus
HDI	hydrodynamic tail vein injection
huFNRG	human hepatocyte chimeric *Fah*$^{-/-}$ NOD *Rag1*$^{-/-}$ *Il2rg*^{null} (mice)
IHI	intrahepatic injection
IRT	mpPHH infection and re-transplantation
mpPHH	mouse-passaged primary human hepatocytes
Mt	mutant
nAb	neutralizing antibody
NRG	NOD *Rag1*$^{-/-}$ *Il2rg*^{null} (mice)
NTCP	sodium taurocholate co-transporting polypeptide
rcccDNA	recombinant covalently closed circular DNA
TRT	mpPHH transfection and re-transplantation
WT	wild type

Suplemental Table 2: HBV Isolate Infection Launch Summary

Isolate	GenBank	TRT			IHI		
		**Success**	**Death**	**Failure**	**Success**	**Death**	**Failure**
D1-1	AB222711	2	4				
D1-2	KC875277			6			
D2-1	MF925358	2		1			
D3	FJ692507	3	1	2	2		
C1-1	DQ089781	1	6	2			
C2-1	KC774182	1	5	3			
C2-2	GQ377617	1	4	1			
C2-3	AP011098	3	2		5		
A1-1	KP168423	1	2				
A1-2	FJ692557	3					
A5	FJ692601	1	1	1			
B2-1	JQ801514	1	2		4		
B2-2	GU815637	1	2				
B3	AP011085		3				
B4	AB073835	1	1	1			
E	KT192626				2	1	
F1	HM585194				3		
DE19 WT	U95551	2					
DE19 PCM		5	1	3	6	1	
DE19 BCPM					3		
C2-3 PCM					4	1	

bioRxiv preprint doi: https://doi.org/10.64898/2026.10.06.756817; this version posted October 8, 2026. The copyright holder for this preprint (which was not certified by peer review) is the author/funder, who has granted bioRxiv a license to display the preprint in perpetuity. It is made available under a CC-BY 4.0 International license.

Isolate	GenBank	TRT			IHI		
		Success	Death	Failure	Success	Death	Failure
C2-3 BCPM					5		
C2-3 DM					3		
B2-1 PCM					2		1
B2-1 BCPM					3		
C_Y_MC	n/a				3		
C_Y_HC					3		
B4 PCM			3				
